# Prevalence of depression and association with all-cause and cardiovascular mortality among individuals with type 2 diabetes: a cohort study based on NHANES 2005–2018 data

**DOI:** 10.1186/s12888-023-04999-z

**Published:** 2023-07-10

**Authors:** Zhen Feng, Wai Kei Tong, Xinyue Zhang, Zhijia Tang

**Affiliations:** 1 Department of Clinical Pharmacy and Pharmacy Administration, School of Pharmacy, Fudan University, 826 Zhangheng Rd, Shanghai, 201203 China; 2 Department of Pharmacy, The Affiliated Hospital of Xuzhou Medical University, No. 99 Huaihai West Road, Xuzhou, 221002 China

**Keywords:** Depression, Diabetes, Mortality, Prevalence, NHANES, Risk factor

## Abstract

**Background:**

Individuals with diabetes have increased risk of depression, but there are limited nationally representative studies on this topic. We aimed to investigate the prevalence and predictors of depression, as well as its impact on all-cause and cardiovascular mortality in adults with type 2 diabetes (T2DM) using a prospective cohort study and a representative sample of the U.S. population.

**Methods:**

We analyzed National Health and Nutrition Examination Survey (NHANES) data from 2005 to 2018 and linked it with the most recent publicly available National Death Index (NDI) data. Individuals aged 20 years or old who had depression measurements were included. Depression was defined as a Patient Health Questionnaire (PHQ-9) score ≥ 10, and categorized into moderate (10–14 points) and moderately severe to severe (≥ 15 points). Cox proportional hazard models were used to estimate the association between depression and mortality.

**Results:**

Among 5695 participants with T2DM, 11.6% had depression. Depression was associated with female gender, younger age, overweight, lower education, being unmarried, smoking, and a history of coronary heart disease and stroke. During a mean follow-up period of 78.2 months, 1161 all-cause deaths occurred. Total depression and moderately severe to severe depression significantly increased all-cause mortality (adjusted hazard ratio [aHR] 1.36, 95% CI [1.09–1.70]; 1.67 [1.19–2.34]) and non-cardiovascular mortality (aHR 1.36, 95% CI [1.04–1.78]; 1.78, 95% CI [1.20–2.64]), but not cardiovascular mortality. Subgroup analysis showed a significant association between total depression and all-cause mortality in males (aHR 1.46, 95% CI [1.08–1.98]) and those aged 60 years or older (aHR 1.35, 95% CI [1.02–1.78]). Any severity of depression was not significantly associated with cardiovascular mortality in age- or gender- stratified subgroups.

**Conclusions:**

In a nationally representative sample of U.S. adults with T2DM, approximately 10% experienced depression. Depression did not significantly associate with cardiovascular mortality. However, comorbid depression in T2DM patients increased the risk of all-cause and non-cardiovascular mortality. The impact of depression on mortality varied across subgroups. Therefore, healthcare providers should consider incorporating depression screening and management into routine care, especially for subgroups with specific risk factors, due to the increased risk of all-cause mortality in T2DM patients with depression.

**Supplementary Information:**

The online version contains supplementary material available at 10.1186/s12888-023-04999-z.

## Background

Depression, a prevalent and severe mental illness characterized by sadness, loss of interest or pleasure, low self-worth, loss of sleep or appetite, tiredness, and difficulty concentrating [1], affects more than 300 million people of all ages globally [2], and is associated with significant social and economic consequences, including lost productivity and expanded healthcare costs [3, 4]. Between 1990 and 2019, depression was one of the primary factors impacting disability-adjusted life-years (DALYs) and was the leading cause of death worldwide [5].

Depression is closely associated with chronic diseases, such as type 2 diabetes (T2DM), which has been linked to a higher prevalence of depressive symptoms [6–11]. For instance, the prevalence of clinically relevant depression (defined as a Patient Health Questionnaire [PHQ-9] score ≥ 10) and clinically significant depression (PHQ-9 score ≥ 15) in U.S. adults with T2DM was 10.6% and 4.2%, respectively, between 2005 and 2012, with no significant changes over time [12]. Furthermore, a bidirectional relationship has been established between T2DM and depression. The risk of incident T2DM was higher in individuals with depressive symptoms. Diabetes, in turn, increases the risk of incident depression and can lead to more severe symptoms [13]. Several factors, such as female gender, low birth weight, adverse childhood events, lifestyle, obesity, cognitive dysfunction, and migration, have been identified to influence the development of comorbid diabetes and depression through multiple mechanisms [14–16].

Individuals with comorbid depression and T2DM have a higher risk of all-cause and cardiac-related death compared to those without depression [17–20]. Multiple studies have demonstrated the negative impact of concomitant T2DM and depression on all-cause mortality [21–24], with remarkably higher risk reported in specific subgroups, such as individuals aged over 75 years (hazard ratio [HR] 6.04) [22], postmenopausal women (HR 5.02) [25], Mexican Americans (odds ratio [OR] 4.04) [26], and individuals with heart failure (HR 3.71) [27]. Other predictive factors include insulin use (HR 2.37), lower limb amputation (HR 1.99), and male gender (HR 1.90) [22].

The mechanisms underlying the increased risk of death in individuals with comorbid depression and diabetes are not fully understood and may be multifactorial. Both physiological and behavioral factors may contribute to it. Hypothalamic-pituitary-adrenal (HPA) axis, sympathetic nervous system (SNS), pro-inflammatory cytokines, and platelet dysfunction, have been suggested to induce CVD and mortality in individuals with comorbid depression [21, 25, 28, 29]. In addition, chronically ill patients with comorbid depression and diabetes tend to have unhealthy behaviors, such as unhealthy diet and lifestyle, low physical activity, smoking [24], as well as poor self-management [30], and non-adherence [31], all of which can worsen short- and long-term clinical outcomes.

However, due to limitations such as small sample size, limited age range, diverse screening tools, and short follow-up time, it is difficult to identify which factors play a major role in increasing this risk. Nor is there established evidence for predicting the comorbid occurrence of diabetes and depression in large populations. One meta-analysis showed that comorbid depression significantly increased the risk of death in people with diabetes; however, the included studies were either from the community or from hospitals or other primary care settings, and no national samples were surveyed [32]. Also, it remains unclear whether the findings are consistent across subpopulations. Considering the adverse health outcomes of these two diseases, there is an urgent need to identify vulnerable subgroups and provide personalized treatment. Moreover, identifying factors linking comorbid depressive symptoms and subsequent death can be an important strategy for diabetes management. In this study, we took advantage of data from the National Health and Nutrition Examination Survey (NHANES), a larger nationwide sample size, to obtain comprehensive results. The objective of this study was to investigate the impact of depression on all-cause and cardiovascular mortality in adults with T2DM, as well as the prevalence and predictors of depression in this population.

## Methods

### Data source and study population

This study used data from the NHANES conducted between 2005 and 2018. The NHANES is a cross-sectional survey that utilizes a complex, multistage, stratified, clustered probability design [33]. It is a nationally representative sample of the civilian non-institutionalized U.S. population.

Initially, the study included 39749 adults over the age of 20. Of these, 6766 were diagnosed with diabetes. We excluded pregnant women (n = 12), individuals with probable type 1 diabetes (n = 216), those with no measures of depression (n = 835), and those lost to follow-up (n = 8). Ultimately, 5695 eligible subjects were included in this analysis.

### Assessment of T2DM

In this study, we defined T2DM as either a self-reported diagnosis by a physician, plasma HbA1c ≥ 6.5%, or fasting plasma glucose ≥ 7.0 mmol/L [34]. We excluded subjects who had possible type 1 diabetes, were diagnosed with diabetes before the age of 30, started insulin within two years of diagnosis, or were currently using insulin [35, 36]. Physician-diagnosed diabetes records were obtained during household interviews.

### Measurement of depression

The PHQ-9 questionnaire has been used to screen depression in NHANES since 2005. It is a reliable and internally consistent assessment tool recommended for measuring depression severity [36, 37]. The PHQ-9 total score ranges from 0 to 27, with a score of ≥ 10 indicating depression. We further subdivided the PHQ-9 scores into three categories: 10–14 points (moderate), 15–19 points (moderately severe), and ≥ 20 points (severe) [17]. Due to the relatively low prevalence of severe depression, we combined the “moderately severe” and “severe” categories into “moderately severe to severe depression” [17].

### Ascertainment of mortality data

We used the Public-use Linked Mortality Files (LMF) from the National Death Index (NDI) as of December 31, 2019, to ascertain mortality data [5]. Causes of death were coded according to the International Classification of Diseases 10th Revision (ICD-10). The endpoints included mortality from all causes, cardiovascular and non-cardiovascular mortality. The deaths related to cardiovascular disease were classified as those coded I00-I09, I11, I13, and I20-I51, while non-cardiovascular deaths encompassed all other causes.

### Covariates

We included several variables as covariates in our analysis, including age, gender, body mass index (BMI), race/ethnicity, education level, marital status, smoking status, and medical history of hypertension, coronary heart disease (CHD), and stroke. Marital status was categorized as cohabitated (including married and living with a partner) and solitary (including widowed, divorced, separated, and never married). Smoking status was categorized as never smokers and smokers (including current smoking and smoking history). Hypertension was defined as systolic blood pressure (SBP) ≥ 130 mmHg, diastolic blood pressure (DBP) ≥ 80 mmHg, self-reported hypertension, or use of antihypertensive medications, with SBP and DBP calculated as the average of all available blood pressure measurements. Both CHD and stroke were self-reported.

### Statistical analysis

For the descriptive statistics, we used mean (standard error, SE) for continuous variables and the number (weighted percentage, %) for categorical variables. We described the baseline depressive status using the PHQ-9 score and compared differences between groups using the Chi-square test. To identify risk factors for depression in patients with T2DM, logistic regression was conducted to calculate odds ratios (ORs). Adjusted ORs were obtained through multivariate regression analyses using the selection methods to account for potential confounding variables. Confounding was assessed by entering potential cofounders into a logistic regression model one at a time. The selection of relevant covariates was based on their relationship with the outcomes of interest, such as baseline characteristics and disease history. Follow-up time was calculated using person-months from the date of interview to the date of death or the end of the follow-up period (December 31, 2019), whichever came first. We calculated the incidence rates of all-cause and cardiovascular mortality by dividing the number of events by the follow-up person-years. Multivariate Cox proportional hazards models were used to estimate HRs with 95% confidence intervals (CIs) of all covariates.

All data were weighted to ensure an appropriate estimate and representative of the total civilian non-institutionalized U.S. population. All statistical analyses were performed using the Survey package in R statistical software (version R 4.2.1 for macOS). A two-sided *p* < 0.05 was considered to be statistically significant.

## Results

### Baseline characteristics of study population

Table 1 showed the baseline characteristics of participants based on depression status from NHANES. We included 5695 participants with T2DM, of whom 722 (weighted percentage 11.6%) had depression. The weighted mean age was 57.4 ± 0.7 years in the depression group and 60.3 ± 0.3 years in the non-depression group. Depressed individuals were more likely to be younger, female, overweight, less educated, solitary, current smoker, and have a history of stroke than those without depression (all *p* values < 0.05).

**Table 1:** Baseline characteristics of study population, NHANES 2005–2018

Characteristics	Total (n = 5695)	Non-depression (n = 4973)	Depression (n = 722)	P value
Age, years	59.9 ± 0.3	60.3 ± 0.3	57.4 ± 0.7	< 0.001
< 60	2156 (46.3)	1826 (45.2)	330 (54.1)	0.004
≥ 60	3539 (53.7)	3147 (54.8)	392 (45.9)	
Gender				
Male	2994 (52.2)	2716 (54.4)	278 (35.3)	< 0.001
Female	2701 (47.8)	2257 (45.6)	444 (64.7)	
BMI, kg/m²				
< 25	717 (10.7)	647 (11.0)	70 (7.7)	< 0.001
25–30	1580 (25.3)	1446 (26.8)	134 (14.1)	
≥ 30	3295 (64.0)	2795 (62.2)	500 (78.2)	
Race/ethnicity				
Non-Hispanic white	1978 (61.3)	1717 (61.5)	261 (59.2)	0.718
Non-Hispanic black	1522 (15.2)	1351 (15.1)	171 (15.9)	
Mexican American	1047 (9.8)	909 (9.7)	138 (10.4)	
Other races	1148 (13.8)	996 (13.7)	152 (14.5)	
Education level				
Below high school	1942 (23.4)	1608 (21.9)	334 (35.0)	< 0.001
High school/Some college	2864 (56.6)	2528 (56.7)	336 (56.3)	
College graduate or above	881 (20.0)	831 (21.4)	50 (8.7)	
Marital status				
Cohabitated	3378 (63.8)	3040 (65.4)	338 (51.1)	< 0.001
Solitary	2311 (36.2)	1928 (34.6)	383 (48.9)	
Smoking status				
Never smoker	2817 (48.7)	2523 (49.8)	294 (39.6)	< 0.001
Past smoker	1949 (35.4)	1716 (35.9)	233 (31.7)	
Current smoker	925 (15.9)	730 (14.2)	195 (28.7)	
Drinking status				
Non-drinker	2894 (98.6)	2577 (98.7)	317 (97.2)	0.155
Drinker	54 (1.4)	45 (1.3)	9 (2.8)	
Hypertension	4589 (80.3)	944 (80.1)	600 (82.0)	0.356
Coronary heart disease	572 (11.2)	481 (10.8)	91 (14.5)	0.092
Stroke	497 (8.1)	401 (7.6)	96 (12.4)	< 0.001

Continuous variables were presented as mean ± SE. Categorial variables were presented as number and weighted percentage. Abbreviations: BMI body mass index

### Prevalence and risk factors of depression in adults with T2DM

The prevalence of depression in adults with T2DM remained stable between 2005 and 2018, both from an overall and subgroup perspective (*p* > 0.05 for all linear trends, supplementary Table S1). Table 2 suggested that the risk of depression was significantly lower in people aged 60 years and older compared to those under 60 (adjusted OR [aOR] 0.77, 95% CI [0.59-1.00]). High school and college graduate or above education also reduced the probability of depression compared to those with a degree below high school (aOR 0.62, 95% CI [0.48–0.79]; aOR 0.33, 95% CI [0.21–0.51]). In contrast, depression was more prevalent among females (aOR 2.02, 95% CI [1.59–2.56]), individuals with BMI ≥ 30 kg/m² (aOR 1.92, 95% CI [1.33–2.76]), solitary individuals (aOR 1.47, 95% CI [1.13–1.90]), current smokers (aOR 2.41, 95% CI [1.86–3.11]), and those with a history of CHD (aOR 1.69, 95% CI [1.10–2.58]) and stroke (aOR 1.65, 95% CI [1.20–2.29]).

**Table 2:** Odds ratios for depression in participants with different characteristics

Characteristics	Odds ratio (95% CI)	
	Unadjusted	Adjusted
Age, years		
< 60	1.00 (reference)	1.00 (reference)
≥ 60	**0.70 (0.55–0.89)**	**0.77 (0.59-1.00)**
Gender		
Male	1.00 (reference)	1.00 (reference)
Female	**2.18 (1.75–2.72)**	**2.02 (1.59–2.56)**
BMI, kg/m²		
< 25	1.00 (reference)	1.00 (reference)
25–30	0.76 (0.52–1.13)	0.86 (0.57–1.30)
≥ 30	**1.81 (1.29–2.55)**	**1.92 (1.33–2.76)**
Race		
Non-Hispanic white	1.00 (reference)	1.00 (reference)
Non-Hispanic black	1.10 (0.86–1.41)	0.78 (0.60–1.02)
Mexican American	1.12 (0.86–1.45)	0.84 (0.63–1.13)
Other	1.10 (0.80–1.52)	1.09 (0.78–1.51)
Education level		
Below high school	1.00 (reference)	1.00 (reference)
High school/Some college	**0.62 (0.49–0.79)**	**0.62 (0.48–0.79)**
College graduate or above	**0.25 (0.17–0.39)**	**0.33 (0.21–0.51)**
Marital status		
Cohabitated	1.00 (reference)	1.00 (reference)
Solitary	**1.81 (1.43–2.30)**	**1.47 (1.13–1.90)**
Smoking status		
Never smoker	1.00 (reference)	1.00 (reference)
Past smoker	1.11 (0.87–1.42)	1.25 (0.96–1.62)
Current smoker	**2.54 (1.92–3.34)**	**2.41 (1.86–3.11)**
Drinking status		
Non-drinker	1.00 (reference)	1.00 (reference)
Drinker	2.21 (0.72–6.80)	1.80 (0.59–5.50)
Hypertension		
No	1.00 (reference)	1.00 (reference)
Yes	1.13 (0.87–1.45)	1.09 (0.83–1.41)
Coronary heart disease		
No	1.00 (reference)	1.00 (reference)
Yes	1.40 (0.94–2.07)	**1.69 (1.10–2.58)**
Stroke		
No	1.00 (reference)	1.00 (reference)
Yes	**1.73 (1.28–2.35)**	**1.65 (1.20–2.29)**

Multivariate logistic regression adjusted for age, gender, BMI, education level, marital and smoking status, and history of stroke (categorial). Numbers in bold indicated significant findings. Abbreviations: BMI body mass index; CI confidence interval

### Association of depression with all-cause and cardiovascular mortality in adults with T2DM

The study found that during an average follow-up period of 78.2 months (depression group: 75.3 months; non-depression group: 78.5 months), there were 1161 deaths, including 323 cardiovascular deaths. The incidence rates of all-cause and CVD mortality among participants with depression were higher than those without depression (Table 3).

After adjusting for covariates, both total depression and moderately severe to severe depression demonstrated a significant association with an increased risk of all-cause death when compared to individuals without depression (adjusted HR [aHR] 1.36, 95% CI [1.09–1.70] and 1.67, 95% CI [1.19–2.34], respectively). Additionally, total depression and moderately severe to severe depression were found to increase the risk of non-cardiovascular mortality among adults with T2DM (aHR 1.36, 95% CI [1.04–1.78] and 1.78, 95% CI [1.20–2.64], respectively). However, no significant association was found between any severity of depression and cardiovascular mortality (Table 3). These findings suggest that depression is a risk factor for all-cause mortality in adults with T2DM but may not be associated with cardiovascular mortality.

**Table 3:** Hazard ratios for all-cause, cardiovascular and non-cardiovascular mortality in participants with depression, stratified by severity

Depression status	Cases/person-years	Incidence per 1000 person-years	Unadjusted HR (95% CI)	Adjusted HR (95% CI)
*All-cause mortality*				
Non-depression	992/33042	27.05	1.00 (reference)	1.00 (reference)
Total depression	169/4610	33.39	**1.24 (1.02–1.53)**	**1.36 (1.09–1.70)**
Moderate	100/2690	31.01	1.15 (0.91–1.46)	1.20 (0.95–1.52)
Moderately severe to severe	69/1921	37.53	1.40 (0.99–1.99)	**1.67 (1.19–2.34)**
*Cardiovascular mortality*				
Non-depression	274/33042	7.40	1.00 (reference)	1.00 (reference)
Total depression	49/4610	9.13	1.24 (0.83–1.85)	1.34 (0.90–2.02)
Moderate	28/2690	8.92	1.20 (0.76–1.92)	1.34 (0.84–2.12)
Moderately severe to severe	21/1921	9.49	1.30 (0.71–2.36)	1.36 (0.70–2.66)
Non-CVD mortality				
Non-depression	718/33042	19.64	1.00 (reference)	1.00 (reference)
Total depression	120/4610	24.26	1.25 (0.99–1.57)	**1.36 (1.04–1.78)**
Moderate	72/2690	22.08	1.14 (0.86–1.49)	1.15 (0.86–1.55)
Moderately severe to severe	48/1921	28.04	1.44 (0.97–2.14)	**1.78 (1.20–2.64)**

Adjustment for age, gender, body mass index, education level, race, marital and smoking status, and history of hypertension, coronary heart disease, and stroke. Incidence and correlation were weighted. Numbers in bold indicated significant findings. Abbreviations: HR hazard ratio; CI confidence interval; CVD cardiovascular disease

### Hazard ratios for all-cause and cardiovascular mortality in subgroups

We conducted a subgroup analysis to address the age- and gender-specific considerations regarding the impact of depression on mortality risk in patients with T2DM (Table 4). The association between total depression and all-cause mortality was only significant in individuals aged 60 years or older (aHR 1.35, 95% CI [1.02–1.78]) and males (aHR 1.46, 95% CI [1.08–1.98]). Furthermore, moderately severe to severe depression significantly increased the risk of all-cause death in both subgroups compared to those without depressive symptoms (aHR 1.69, 95% CI [1.10–2.58]; aHR 1.75, 95% CI [1.06–2.90]). No remarkable association with cardiovascular mortality was observed in any age or gender subgroups, consistent with the overall results. When considering non-cardiovascular mortality, the association between total depression and non-cardiovascular mortality was significant only in people aged 60 years or older (aHR 1.40, 95% CI [1.00-1.96]). Additionally, moderately severe to severe depression significantly increased the risk of non-cardiovascular death in people aged 60 years or older (aHR 1.87, 95% CI [1.15–3.05]) and males (aHR 1.86, 95% CI [1.02–3.42]).

**Table 4:** Adjusted hazard ratios for all-cause, cardiovascular and non-cardiovascular mortality in participants with different severity of depression, stratified by age and gender

Characteristics	Adjusted HR (95% CI)		
	Total depression	Moderate depression	Moderately severe to severe depression
*All-cause mortality*			
Age, years			
< 60	1.31 (0.83–2.06)	1.17 (0.63–2.17)	1.53 (0.87–2.70)
≥ 60	**1.35 (1.02–1.78)**	1.19 (0.88–1.61)	**1.69 (1.10–2.58)**
Gender			
Male	**1.46 (1.08–1.98)**	1.34 (0.93–1.93)	**1.75 (1.06–2.90)**
Female	1.32 (0.98–1.78)	1.15 (0.84–1.56)	1.64 (0.96–2.78)
*Cardiovascular mortality*			
Age, years			
< 60	1.65 (0.72–3.78)	1.63 (0.54–4.95)	1.70 (0.68–4.23)
≥ 60	1.21 (0.74–1.99)	1.20 (0.68–2.11)	1.23 (0.51–2.95)
Gender			
Male	1.54 (0.95–2.50)	1.59 (0.94–2.70)	1.43 (0.55–3.72)
Female	1.25 (0.73–2.15)	1.19 (0.66–2.15)	1.37 (0.55–3.41)
*Non-CVD mortality*			
Age, years			
< 60	1.23 (0.74–2.05)	1.07 (0.53–2.14)	1.49 (0.77–2.89)
≥ 60	**1.40 (1.00-1.96)**	1.19 (0.82–1.72)	**1.87 (1.15–3.05)**
Gender			
Male	1.43 (0.95–2.15)	1.25 (0.75–2.06)	**1.86 (1.02–3.42)**
Female	1.35 (0.95–1.91)	1.13 (0.79–1.61)	1.74 (0.94–3.20)

Reference: non-depression group. Adjustment for age, gender, body mass index, education level, race, marital and smoking status, and history of hypertension, coronary heart disease, and stroke. Abbreviations: HR hazard ratio; CI confidence interval; CVD cardiovascular disease. Numbers in bold indicated significant findings

## Discussion

This is a large-scale, prospective, long-term follow-up, nationally representative cohort study. We estimated the prevalence of depression and its predictors, as well as the effects of depression on all-cause, cardiovascular mortality and non-cardiovascular mortality in patients with T2DM, at the overall and subgroup levels, which are rarely reported.

### Prevalence of depression and its predictors in individuals with T2DM

In this study, we estimated the overall prevalence of depression, as measured by a PHQ-9 score ≥ 10, was 11.6% in T2DM patients over 20 years of age. The National Survey on Drug Use and Health (NSDUH) found that the prevalence of depression was 7.1% in all U.S. adults. Consistently, it was thought that comorbid T2DM increased the risk of depression and may lead to a more severe course of depression [16]. This is consistent with previous research but with stability over a longer period [12, 38]. However, caution must be exercised in generalizing the findings to other countries due to possible variations in participant samples, study sites, and methods of assessing depression [39, 40].

Therefore, it is important to explore the prevalence of depression and its predictors across different populations and setting to better understand the burden of this comorbidity. The 2015–2018 NHANES data showed an increased prevalence of depression in almost all subgroups of people with T2DM [11, 18]. In this study, we observed that sociodemographic variables such as younger age, female gender, lower educational level, being overweight or unmarried, smoking, and a history of chronic medical conditions were major predictors of depression among patients with T2DM. These findings highlight the need for targeted interventions to address the unique risk factors.

Younger people with T2DM tend to be more vulnerable to depression due to their increased susceptibility to negative life events, such as being diagnosed with a chronic disease, which may lead to worse moods, more distress, and more depression than older adults [38, 41]. Additionally, they may lack the coping skills and experience to effectively manage these situations and help reduce the perception of harmful consequences [7]. These findings suggest the importance of targeting depression screening and intervention efforts toward younger T2DM patients, in order to mitigate the potential negative impact of depression on their overall health outcomes.

Women in this study were twice as likely as men to suffer from depression (15.6% vs. 7.8%; aOR 2.02), consistent with prior evidence [38, 42, 43]. Higher screening rates and women’s inherent predisposition to depression may partly explain this gender difference [44]. We also observed that depression was more prevalent among individuals with lower levels of education, which is consistent with previous research indicating the protective effect of higher education against anxiety and depression resulting from improvements in financial hardship, physical health, and occupational restrictions [45]. Regarding marital and smoking status, a meta-analysis suggests that single or unmarried people with depression have worse prognoses [46]. The association between smoking and mental illness has long been suspected, but causality and underlying mechanisms remain to be studied [47]. Both were consistent with our results.

In addition, many studies have shown that depression frequently coexists with chronic diseases [3, 48–52]. Our findings suggested an increased risk of depression in diabetic patients with CHD (aOR = 1.69) and stroke (aOR = 1.65). These findings emphasize the importance of identifying and treating depression in patients with T2DM, especially those with comorbidities.

### Impact and risk factors on mortality of comorbid T2DM and depression

In this study, we observed that patients with T2DM and depression, especially moderately severe to severe depression, had higher all-cause and non-cardiovascular mortality than those without depression, while depression of any severity had no significant effect on cardiovascular mortality. Our results were consistent with most previous studies on all-cause mortality but differed on cardiovascular mortality [21, 53–55]. Differences in participant characteristics, diagnostic criteria, duration of follow-up, treatment regimens, and potential confounders in the final model may be underlying the reason.

Regarding the risk factors for mortality, our subgroup analysis revealed that age and gender played different roles. The association between total depression and all-cause mortality was more pronounced among individuals aged 60 years or older, while it was not statistically significant in younger subjects. The reason for this disparity is unknown, and further studies are needed. Moreover, although women were found to be at greater risk of developing depression, subgroup analysis demonstrated that depression had a greater impact on all-cause mortality in men. Previous studies have consistently suggested that poor psychological status has a more profound effect on mortality in men [19, 56], possibly because men are less likely to seek help for mental health issues than women [57–59]. Another pathophysiological explanation for this is that men are more susceptible to lipid oxidative damage caused by depression, which could be a key event in the etiology of atherosclerosis and CVD [19, 60].

As the severity of depression escalates, its impact on mortality becomes more prominent, both overall and within subgroups. To our knowledge, few studies have explored the effect of depression severity on mortality, making our study particularly noteworthy. However, due to the small sample size of some subgroups, our study may still be underpowered to detect statistically significant differences. Further studies with larger samples and longer follow-ups are warranted to confirm these findings.

As NHANES did not include prescribing details, it was unclear whether antidepressants helped reduce mortality in our study. Considering the close association of depression with poor self-management, interventions targeting self-care behaviors may be beneficial. Clinicians should also pay special attention to suicidal attempts, which are a common cause of death, particularly among older adults with depression [61].

This study had several limitations that should be taken into consideration. First, we relied on self-reported demographic data and the PHQ-9 questionnaire, which was not validated and may have resulted in the misclassification of participants. Additionally, due to a lack of information, there may be unmeasured confounding factors that we were unable to adjust for, such as the family history of depression and diabetes, glycemic control, medication use, disease duration, behavioral changes, and the presence of other comorbidities. Lastly, the timeframe of our study did not include the COVID-19 pandemic, which has had a substantial impact on people’s psychological well-being.

## Conclusions

In conclusion, we reported the prevalence of depression in T2DM patients using the NHANES 2005–2018 dataset and identified several predictors of depression. We found that the presence of comorbid depression in T2DM patients is associated with an increased mortality risk relative to those without depression. Specifically, depression was associated with more than 30% increased risk of all-cause and non-cardiovascular death, while no significant association was observed with cardiovascular mortality. Furthermore, the impact of depression on mortality varied depending on gender and age, with older patients and males with T2DM experiencing a high risk of all-cause and non-cardiovascular death. These findings emphasize the importance of mental health awareness and management for patients with T2DM, particularly among subgroups with specific risk factors.

## Electronic supplementary material

Below is the link to the electronic supplementary material.

Supplementary Material 1

## Data Availability

The datasets generated and/or analyzed during the current study are publicly available from CDC National Center for Health Statistics (https://www.cdc.gov/nchs/nhanes/index.htm).

## Abbreviations

BMI: body mass index
CHD: coronary heart disease
CI: confidence interval
DALYs: disability-adjusted life-years
DBP: diastolic blood pressure
HPA: Hypothalamic-pituitary-adrenal
HR: hazard ratio
ICD-10: International Classification of Diseases 10th Revision
LMF: Linked Mortality Files
NDI: National Death Index
NHANES: National Health and Nutrition Examination Survey
NSDUH: National Survey on Drug Use and Health
PHQ-9: Patient Health Questionnaire
SBP: systolic blood pressure
SE: standard error
SNS: sympathetic nervous system
