# Supplementary material for: Prevalence of depression and association with all-cause and cardiovascular mortality among individuals with type 2 diabetes: a cohort study based on NHANES 2005–2018 data
Source: BMC Psychiatry. 2023 Jul 10;23:490. doi: 10.1186/s12888-023-04999-z (PMC10331954; doi:10.1186/s12888-023-04999-z)
Supplement: Supplementary file 1 — Supplementary Material 1 [file 12888_2023_4999_MOESM1_ESM.pdf]

Supplemental Table S1. Prevalence of depression among adults with type 2 diabetes in the U.S. by characteristics, NHANES 2005-2018.

Characteristics	2005-2018 % (95% CI)	2005-2006 % (95% CI)	2007-2008 % (95% CI)	2009-2010 % (95% CI)	2011-2012 % (95% CI)	2013-2014 % (95% CI)	2015-2016 % (95% CI)	2017-2018 % (95% CI)	P value for trend
**Total depression (PHQ-9 score: ≥10)**									
Overall	11.6 (10.5-12.6)	9.1 (6.8-11.4)	12.1 (9.2-15.0)	11.9 (9.3-14.5)	12.1 (9.0-15.2)	13.4 (9.5-17.2)	11.3 (8.7-13.8)	10.7 (8.8-12.7)	0.743
Age, years									
<60	13.5 (11.7-15.4)	11.6 (6.5-16.7)	15.1 (10.4-19.9)	16.0 (10.8-21.2)	14.2 (10.2-18.2)	14.8 (7.2-22.3)	12.8 (8.0-17.5)	10.8 (6.4-15.2)	0.211
≥60	9.9 (8.5-11.3)	6.8 (2.8-10.8)	9.0 (5.1-12.9)	8.7 (5.6-11.9)	10.2 (6.1-14.2)	12.2 (6.8-17.6)	10.0 (5.9-14.0)	10.7 (7.5-13.8)	0.100
Gender									
Male	7.8 (6.7-9.0)	8.5 (3.3-13.8)	8.2 (5.6-10.9)	8.5 (6.1-10.8)	6.1 (2.9-9.2)	8.0 (4.0-11.9)	6.1 (3.5-8.7)	9.6 (6.2-12.9)	0.971
Female	15.6 (13.8-17.5)	9.7 (6.4-12.9)	16.1 (11.2-21.0)	15.8 (10.4-21.3)	17.9 (11.8-24.1)	19.5 (13.9-25.2)	17.5 (12.6-22.4)	12.0 (7.0-17.1)	0.536
Race/ethnicity									
Non-Hispanic White	11.2 (9.5-12.8)	9.1 (5.3-12.9)	11.6 (7.3-15.8)	10.4 (6.2-14.6)	10.3 (5.9-14.7)	12.7 (6.4-18.9)	12.2 (6.8-17.6)	11.0 (7.8-14.3)	0.422
Non-Hispanic Black	12.1 (10.4-13.9)	11.6 (8.9-14.3)	12.6 (8.7-16.4)	17.0 (9.7-24.2)	12.9 (8.6-17.3)	15.6 (9.4-21.7)	6.9 (3.5-10.4)	9.8 (5.3-14.2)	0.063
Other races	12.2 (10.4-14.0)	6.8 (2.0-11.5)	13.6 (7.6-19.6)	12.8 (8.2-17.3)	15.5 (8.2-22.9)	14.0 (10.2-17.8)	11.4 (7.9-14.8)	10.5 (5.9-15.2)	0.769
Education level									
<High school	17.3 (15.0-19.5)	8.3 (3.5-13.1)	15.3 (10.7-19.8)	21.8 (16.3-27.4)	20.8 (10.9-30.8)	22.0 (15.0-29.1)	13.1 (8.8-17.3)	18.1 (11.2-25.0)	0.151
≥High school graduate	9.8 (8.6-11.0)	9.4 (6.2-12.5)	10.8 (6.8-14.9)	8.1 (6.0-10.3)	8.9 (5.9-11.9)	11.0 (6.5-15.5)	10.7 (6.9-14.5)	9.1 (6.8-11.5)	0.490
Marital status									
Married or living with partner	9.3 (7.8-10.7)	8.5 (4.9-12.2)	10.5 (6.1-15.0)	10.3 (6.2-14.4)	7.5 (4.4-10.6)	12.1 (6.0-18.2)	9.7 (6.0-13.4)	6.8 (3.6-9.9)	0.430
Unmarried	15.6 (13.8-17.4)	10.3 (7.2-13.4)	14.9 (10.1-19.6)	14.8 (10.0-19.6)	18.8 (13.1-24.4)	15.4 (10.7-20.1)	14.0 (8.4-19.7)	18.9 (13.7-24.1)	0.076
Smoke									

never-smoker	9.4 (7.9-10.9)	8.0 (3.9-12.2)	7.0 (3.5-10.5)	10.4 (6.7-14.0)	11.1 (5.9-16.2)	11.6 (6.9-16.2)	8.4 (4.5-12.3)	9.2 (5.1-13.3)	0.592
smoker	13.6 (12.1-15.1)	10.2 (5.1-15.3)	16.6 (12.0-21.2)	13.3 (10.1-16.4)	13.2 (9.5-16.9)	15.1 (9.3-20.8)	13.7 (10.4-17.1)	12.5 (8.4-16.5)	0.997
Hypertension, no	10.7 (8.4-12.9)	6.1 (1.2-11.0)	12.3 (3.4-21.2)	12.6 (9.3-16.0)	16.8 (9.1-24.4)	11.4 (3.8-19.1)	9.6 (4.5-14.6)	6.2 (2.5-9.8)	0.390
Hypertension, yes	11.9 (10.7-13.0)	9.8 (7.5-12.2)	12.0 (8.9-15.2)	11.8 (8.7-14.8)	11.2 (7.5-14.8)	14.1 (10.0-18.2)	11.9 (8.1-15.7)	11.7 (9.4-14.0)	0.400
Coronary heart disease, no	10.7 (8.4-12.9)	9.3 (6.8-11.7)	12.1 (8.5-15.7)	10.9 (7.5-14.3)	10.9 (7.9-13.8)	12.5 (8.4-16.7)	11.2 (8.1-14.2)	10.3 (7.9-12.8)	0.844
Coronary heart disease, yes	11.9 (10.7-13.0)	7.8 (0.9-14.7)	12.2 (2.6-21.8)	18.3 (4.0-32.7)	17.6 (0-35.6)	20.2 (8.1-32.2)	14.3 (0-29.7)	13.0 (2.8-23.3)	0.624
Stroke, no	11.0 (9.9-12.1)	8.7 (6.3-11.1)	11.6 (8.4-14.8)	11.5 (8.4-14.7)	11.3 (7.8-14.9)	13.0 (8.7-17.3)	11.2 (8.4-14.0)	9.3 (7.0-11.7)	0.961
Stroke, yes	17.6 (13.6-21.6)	12.7 (5.9-19.5)	17.1 (8.0-26.3)	16.4 (8.5-24.3)	23.5 (7.2-39.8)	17.8 (5.1-30.5)	12.3 (5.6-18.9)	22.9 (9.9-35.9)	0.342
**Moderate depression (PHQ-9 score: 10-14)**									
Overall	7.3 (6.4-8.2)	6.0 (3.7-8.3)	8.4 (5.2-11.7)	6.8 (5.1-8.6)	6.7 (4.6-8.8)	8.4 (4.8-11.9)	6.5 (4.2-8.8)	7.8 (5.8-9.8)	0.702
Age, years									
<60	8.3 (6.8-9.7)	6.5 (3.7-9.4)	10.5 (5.6-15.4)	9.0 (5.8-12.1)	8.1 (4.5-11.8)	8.2 (2.6-13.8)	7.7 (4.1-11.3)	7.9 (4.3-11.6)	0.700
≥60	6.4 (5.2-7.7)	5.6 (2.3-8.8)	6.3 (2.6-10.1)	5.2 (3.6-6.8)	5.4 (2.4-8.3)	8.5 (3.4-13.6)	5.5 (2.8-8.3)	7.7 (4.2-11.2)	0.317
Gender									
Male	5.4 (4.4-6.4)	4.9 (1.7-8.1)	5.5 (2.6-8.4)	5.6 (3.2-8.0)	3.7 (2.0-5.4)	6.6 (2.4-10.9)	3.5 (1.2-5.7)	7.6 (4.6-10.5)	0.454
Female	9.4 (7.7-11.0)	7.1 (3.6-10.6)	11.5 (6.4-16.6)	8.3 (5.6-10.9)	9.6 (5.7-13.5)	10.4 (5.0-15.7)	10.2 (6.2-14.3)	8.1 (2.9-13.2)	0.969
Race/ethnicity									
Non-Hispanic White	7.4 (6.0-8.8)	5.8 (2.0-9.5)	9.2 (4.8-13.6)	6.0 (3.4-8.5)	6.1 (2.9-9.2)	8.8 (3.3-14.3)	7.4 (3.3-11.4)	7.9 (4.3-11.4)	0.624
Non-Hispanic Black	6.9 (5.5-8.4)	7.6 (4.2-11.0)	5.9 (3.2-8.6)	9.9 (5.1-14.7)	5.2 (2.4-7.9)	9.4 (3.8-14.9)	3.9 (1.13-6.69)	7.8 (2.7-12.9)	0.734
Other races	7.2 (5.8-8.6)	5.4 (1.4-9.4)	8.4 (3.5-13.2)	7.3 (2.4-12.1)	9.0 (4.9-13.2)	6.7 (4.6-8.8)	6.0 (3.4-8.6)	7.6 (2.9-12.3)	0.939
Education level									

<High school	9.3 (7.8-10.8)	5.8 (2.1-9.5)	9.0 (5.2-12.7)	11.0 (7.4-14.6)	9.8 (5.7-14.0)	13.0 (6.0-20.1)	5.2 (3.2-7.2)	11.4 (6.6-16.2)	0.440
≥High school graduate	6.6 (5.6-7.7)	6.1 (2.9-9.3)	8.2 (4.3-12.2)	5.3 (3.5-7.1)	5.5 (3.2-7.9)	7.1 (3.3-10.9)	6.8 (3.8-9.9)	6.9 (4.7-9.1)	0.797
Marital status									
Married or living with partner	5.9 (4.7-7.1)	5.4 (2.6-8.2)	7.4 (3.1-11.7)	6.4 (3.4-9.4)	3.6 (1.0-6.2)	7.7 (2.7-12.8)	5.8 (2.7-8.8)	5.0 (2.4-7.7)	0.627
Unmarried	9.8 (8.3-11.3)	7.3 (3.1-11.5)	10.2 (5.6-14.9)	7.7 (4.8-10.6)	11.1 (6.6-15.7)	9.5 (5.6-13.3)	7.9 (4.4-11.3)	13.5 (8.4-18.7)	0.186
Smoke									
never-smoker	5.7 (4.5-7.0)	4.6 (1.5-7.7)	4.8 (2.0-7.7)	5.4 (2.6-8.1)	6.1 (1.8-10.4)	6.7 (2.6-10.7)	5.1 (1.8-8.5)	6.7 (3.5-10.0)	0.314
smoker	8.8 (7.5-10.0)	7.5 (4.1-10.8)	11.6 (6.7-16.5)	8.1 (6.1-10.1)	7.3 (5.3-9.3)	9.9 (4.8-15.1)	7.7 (5.4-10.1)	9.0 (5.4-12.6)	0.781
Hypertension, no	6.0 (4.1-8.0)	1.4 (0-3.4)	9.8 (1.5-18.1)	6.5 (2.8-10.2)	10.9 (2.8-19.0)	5.5 (0-11.0)	6.1 (1.9-10.3)	1.7 (0-4.0)	0.290
Hypertension, yes	7.6 (6.7-8.6)	7.2 (4.4-10.0)	8.0 (5.0-11.0)	7.0 (4.6-9.3)	5.8 (3.6-8.0)	9.3 (5.5-13.0)	6.7 (4.0-9.4)	9.0 (6.8-11.3)	0.379
Coronary heart disease, no	7.1 (6.1-8.0)	6.0 (3.5-8.4)	8.4 (4.9-11.9)	6.4 (4.3-8.5)	5.8 (4.3-7.2)	8.0 (4.1-11.9)	6.6 (4.4-8.9)	7.8 (5.5-10.1)	0.622
Coronary heart disease, yes	8.6 (5.2-11.9)	6.2 (0-14.0)	8.7 (0-17.6)	9.9 (0.5-19.2)	9.8 (0-23.3)	10.8 (0-22.3)	6.5 (0.3-12.7)	7.9 (0.1-15.7)	0.973
Stroke, no	6.9 (6.0-7.8)	5.6 (3.3-8.0)	8.1 (4.9-11.4)	6.4 (4.4-8.4)	6.6 (4.1-9.0)	8.4 (5.1-11.8)	6.5 (4.2-8.8)	6.6 (4.4-8.8)	0.945
Stroke, yes	10.8 (7.3-14.3)	9.0 (2.1-15.8)	11.6 (2.7-20.6)	11.9 (5.3-18.6)	8.6 (0-17.5)	7.8 (0-18.1)	6.7 (0.2-13.3)	17.6 (5.4-29.8)	0.489
**Moderately severe to severe depression (PHQ-9 score: ≥15)**									
Overall	4.3 (3.7-4.9)	3.1 (0.2-5.9)	3.7 (2.9-4.5)	5.1 (3.3-6.8)	5.4 (3.2-7.6)	5.0 (3.7-6.3)	4.7 (2.9-6.6)	2.9 (1.8-4.1)	0.958
Age, years									
<60	5.2 (4.2-6.3)	5.1 (0-10.8)	4.7 (3.2-6.1)	7.0 (3.4-10.6)	6.1 (3.0-9.2)	6.6 (3.4-9.7)	5.1 (2.3-7.8)	2.9 (1.4-4.4)	0.333
≥60	3.5 (2.6-4.3)	1.2 (0-2.6)	2.6 (1.7-3.6)	3.6 (1.5-5.7)	4.8 (2.4-7.2)	3.7 (2.3-5.1)	4.5 (0.6-8.3)	3.0 (1.3-4.7)	0.154
Gender									
Male	2.4 (1.8-3.1)	3.6 (0-7.9)	2.7 (1.7-3.7)	2.9 (1.8-4.0)	2.4 (0.2-4.6)	1.3 (0.5-2.2)	2.7 (0.6-4.7)	2.0 (0.7-3.3)	0.301

Female	6.3 (5.2-7.3)	2.6 (0.7-4.4)	4.7 (2.9-6.4)	7.6 (3.8-11.3)	8.4 (4.3-12.4)	9.2 (6.0-12.4)	7.3 (4.2-10.3)	4.0 (1.8-6.2)	0.229
Race/ethnicity									
Non-Hispanic White	3.8 (2.9-4.7)	3.4 (0-7.7)	2.4 (1.4-3.3)	4.4 (1.8-7.1)	4.2 (1.9-6.5)	3.9 (2.1-5.7)	4.9 (1.5-8.2)	3.1 (1.4-4.9)	0.638
Non-Hispanic Black	5.2 (4.0-6.4)	4.0 (1.1-6.9)	6.7 (3.4-9.9)	7.1 (1.6-12.5)	7.7 (5.1-10.4)	6.2 (2.1-10.3)	3.0 (0.3-5.7)	2.0 (0-4.1)	0.025
Other races	5.0 (4.0-6.0)	1.4 (0-3.0)	5.2 (2.0-8.5)	5.5 (4.4-6.6)	6.5 (1.6-11.3)	7.3 (4.3-10.3)	5.4 (3.4-7.3)	2.9 (1.1-4.8)	0.973
Education level									
<High school	7.9 (6.4-9.5)	2.5 (0.1-4.9)	6.3 (4.1-8.5)	10.8 (6.2-15.4)	11.0 (4.2-17.8)	9.0 (4.1-13.9)	7.9 (4.0-11.7)	6.7 (2.8-10.5)	0.163
≥High school graduate	3.2 (2.5-3.8)	3.3 (0-7.0)	2.6 (1.8-3.4)	2.9 (1.5-4.2)	3.4 (1.8-5.0)	3.9 (2.3-5.5)	3.9 (1.8-5.9)	2.2 (1.1-3.4)	0.943
Marital status									
Married or living with partner	3.4 (2.7-4.1)	3.1 (0-6.5)	3.1 (2.0-4.2)	3.9 (1.4-6.4)	3.9 (1.8-6.0)	4.4 (2.5-6.3)	3.9 (2.1-5.7)	1.8 (0.6-2.9)	0.482
Unmarried	5.8 (4.7-7.0)	3.0 (0.4-5.6)	4.6 (2.8-6.5)	7.1 (3.5-10.7)	7.6 (4.1-11.1)	6.0 (3.4-8.5)	6.2 (2.1-10.2)	5.4 (2.8-7.9)	0.354
Smoke									
never-smoker	3.7 (2.8-4.6)	3.5 (0-8.6)	2.2 (0.8-3.5)	5.0 (2.5-7.4)	5.0 (2.6-7.4)	4.9 (2.6-7.2)	3.3 (1.1-5.5)	2.5 (0.4-4.5)	0.714
smoker	4.9 (4.0-5.7)	2.7 (0.4-5.0)	5.0 (3.5-6.4)	5.2 (2.5-7.8)	5.9 (2.8-9.0)	5.1 (3.3-6.9)	6.0 (3.6-8.4)	3.5 (1.6-5.3)	0.622
Hypertension, no	4.6 (3.2-6.1)	4.7 (0-9.60	2.5 (0.3-4.7)	6.2 (2.7-9.6)	5.9 (0.9-10.8)	5.9 (0.5-11.4)	3.5 (0.1-6.8)	4.4 (1.6-7.2)	0.989
Hypertension, yes	4.2 (3.5-4.9)	2.7 (0-5.3)	4.0 (3.0-5.0)	4.8 (2.7-6.9)	5.4 (2.9-7.8)	4.8 (3.6-6.1)	5.1 (2.2-8.1)	2.7 (1.6-3.8)	0.985
Coronary heart disease, no	4.0 (3.3-4.7)	3.3 (0.1-6.5)	3.6 (2.7-4.5)	4.5 (2.3-6.7)	5.1 (2.7-7.5)	4.5 (3.0-6.0)	4.5 (2.5-6.5)	2.5 (1.6-3.5)	0.691
Coronary heart disease, yes	6.3 (3.7-8.8)	1.6 (0-5.4)	3.5 (0.5-6.4)	8.5 (1.6-15.3)	7.7 (1.4-14.1)	9.4 (4.4-14.5)	7.8 (0-22.3)	5.1 (0.5-9.8)	0.341
Stroke, no	4.1 (3.4-4.7)	3.0 (0-6.2)	3.5 (2.8-4.2)	5.1 (3.1-7.1)	4.8 (2.7-6.9)	4.6 (3.0-6.1)	4.7 (2.7-6.7)	2.7 (1.5-3.9)	0.872
Stroke, yes	6.8 (4.4-9.3)	3.7 (0-8.6)	5.5 (1.6-9.4)	4.5 (0-9.5)	14.9 (.3-29.6)	10.0 (1.5-18.5)	5.6 (0.6-10.5)	5.3 (0.9-9.8)	0.526
